# Effect of Angle of View and Partial Sleep Deprivation on Distance Perception

**DOI:** 10.3389/fpsyg.2020.00201

**Published:** 2020-03-11

**Authors:** Hamza Baati, Hamdi Chtourou, Wassim Moalla, Mohamed Jarraya, Pantelis T. Nikolaidis, Thomas Rosemann, Beat Knechtle

**Affiliations:** ^1^LR18JS01: Education, Motricité, Sport et Santé, High Institute of Sport and Physical Education, University of Sfax, Sfax, Tunisia; ^2^Activité Physique, Sport et Santé, UR18JS01, Observatoire National du Sport, Tunis, Tunisia; ^3^Institut Supérieur du Sport et de l’Education Physique de Sfax, Université de Sfax, Sfax, Tunisia; ^4^Exercise Physiology Laboratory, Nikaia, Greece; ^5^Institute of Primary Care, University Hospital Zurich, Zurich, Switzerland; ^6^Medbase St. Gallen Am Vadianplatz, St. Gallen, Switzerland

**Keywords:** perception, sleep, repeated sprint, angle of view, performance

## Abstract

The present study aimed to investigate the effects of intensive effort on egocentric distance perception according to different angles of view after sleep deprivation at the beginning (SDB) or at the end (SDE) of the night and after a normal sleep night (NNS). Ten male students soccer players (age 22.8 ± 1.3 years; body mass 72.0 ± 10.4 kg; body height 180.0 ± 3.0 cm) performed a repeated cycling (RS) exercise (10 × 6 s maximal cycling with 24 s in between) after SDB, SDE, and NNS. They were asked to estimate three distances (i.e. 15, 25, and 35 m) before and after RS from different angles of view [i.e. in front (0°) and in side (45° left and 45° right)]. For 35 m, distance estimation was better during NNS compared to SDB and SDE for the front and the two side angles either before or after RS (*p* < 0.05). Concerning 25 m, distance estimation was better after compared to before RS for the front angle during the NNS session (*p* < 0.05). For 15 m, distance estimation was better during NNS than SDB and SDE for the front and both side angles after RS (*p* < 0.05). We concluded that partial sleep deprivation negatively affected the estimation of the egocentric distance for the three angles of view either at rest or after RS exercise.

## Introduction

Sports performance is generally related to perceptive and cognitive skills, physiological and energetic capacities, and sleeping (Davenne, 2009; Davranche and McMorris, 2009). In fact, sleep is a reversible state of consciousness characterized by the temporary suspension of perceptual/sensory phenomena and voluntary motor activity that naturally occurs at regular intervals, altering with wakefulness (Liberalesso et al., 2012).

Several studies in this topic reported that the disruption of the sleep–wake cycle, which is one of the main biological circadian rhythms, has various consequences on cognitive and physical performance (Davenne, 2009; Souissi et al., 2012; Jarraya et al., 2014). Although the literature is not consistent about the effect of sleep loss on physical performance (Symons et al., 1988; Souissi et al., 2003, 2008, 2013; HajSalem et al., 2013), it was widely demonstrated that sleep loss affects cognitive functions such as attention (Goel et al., 2013), reaction time (Corsi-Cabrera et al., 1996; Jarraya et al., 2014), alertness (Doran et al., 2001), working memory, abstraction, and decision making (Diekelmann and Born, 2010; Lim and Dinges, 2010). Therefore, psychological states were affected by the sleep disruption (Kaida and Niki, 2014; Short and Louca, 2015) as previously indicated using the profile of mood states (POMS) questionnaire (e.g. anxiety and anger) and the Hooper questionnaire used to assess fatigue, sleep, stress, and muscle soreness (Trabelsi et al., 2018, 2019; Boukhris et al., 2019b).

The optimization of performance in most physical activities and sports depends on the efficiency of physiological and energetic processes as well as the perceptive and cognitive processes (Davranche and McMorris, 2009). In this context, visual perception was assumed to be determined by optical information related to the particular situation of the environment (Witt et al., 2004). During a soccer game, the player needs to estimate distances to pass or intercept the ball and to score using high-intensity physical efforts interspersed by short recovery periods (Spencer et al., 2006; Castagna et al., 2007). However, total or partial sleep deprivation (PSD) when occurring due to a travel camp or any circumstance will influence a player’s performance (Davenne, 2009). In fact, previous to any competition or game, the soccer player’s sleeping period could be reduced or fragmented by anxiety or jet lag, which is considered as one of the main causes of sleep disruption (Davenne, 2009; Edwards and Waterhouse, 2009); due to that, many strategies [e.g. nap (Abdessalem et al., 2019; Boukhris et al., 2019a; Chtourou et al., 2019; Hsouna et al., 2019), caffeine ingestion (Souissi et al., 2014, 2015)] have been proposed to overcome the negative effect of poor sleep. Jet lag is the term applied to a group of symptoms that exist transiently after undergoing a rapid time-zone transition. Also, the term jet lag (i.e. “social jet lag”) refers to a change in sleeping hours (e.g. it is very common to change sleeping hours from workdays to non-working days) (Wittmann et al., 2006; Simmons et al., 2015; Malhotra, 2017). For example, it has been reported that social jet lag affects postural control by a negative effect on brain areas (e.g. thalamus, prefrontal cortex, and cerebellum) (Umemura et al., 2018).

The daytime symptoms include increased fatigue, a loss of concentration and motivation, increased irritability, altered bowel activity, and decreased enjoyment of food (Waterhouse et al., 2005). To measure and spot the changes and variations of sleep, actigraphy provides a non-invasive method to assess sleep–wake cycles over long periods, from days to months. It is based on continuously monitoring body movements and identifying the activity and resting periods (Forner-Cordero et al., 2018).

It is well known that during a normal night of sleep (NNS), a succession of electro-encephalographic changes occur in adults [e.g. changes in the rapid eye movement (REM) (Dement and Kleitman, 1957)], and that sleep deprivation is actively involved in the alteration of the oculomotor system (Lo et al., 2012; Rupp et al., 2012; Tassi et al., 2012). In fact, sleep plays an active role in the processes of adaptation to the reversal of the retinal image and perhaps, by extension, in the processes of information processing in general, such as estimation of the distance from different angles of view (Zimmerman et al., 1970). Indeed, sleep has the function of maintaining the facilitation of binocular coordination, as well as a homeostatic balance in the rate of eye movements throughout the sleep–wake cycle (Dement and Kleitman, 1957).

To the best of our knowledge, no previous study had investigated the effect of distance estimation in different angle of view after PSD. The aim of the present study was, therefore, to examine the effect of PSD and intense physical effort on egocentric distance perception in soccer players according to different angles of view. We hypothesized that distance estimation would be negatively affected by sleep loss and that it is better from the front compared to the side angle of view.

## Materials and Methods

### Participants

Ten male students soccer players (age: 22.8 ± 1.3 years; body mass: 72.0 ± 10.4 kg; body height: 180.0 ± 3.0 cm) volunteered to participate in the experiment. They had at least 8 years of football practice and were pursuing university studies in Sport Sciences. Their average weekly training volume was ∼10 h per week including various physical activities as part of their university courses. The participants had complied with a regular given sleeping schedule. In fact, all participants had normal-to-corrected vision that was checked by a physician. They were classified as “moderate morning type” according to their responses to the Horne and Ostberg (1976) questionnaire. Before the beginning of the study, all the participants were informed about the procedure of the study before giving their consent to participate. The study was approved by the local ethics committee and was conducted according to the Declaration of Helsinki (1975).

### Experimental Design

**FIGURE 1:**
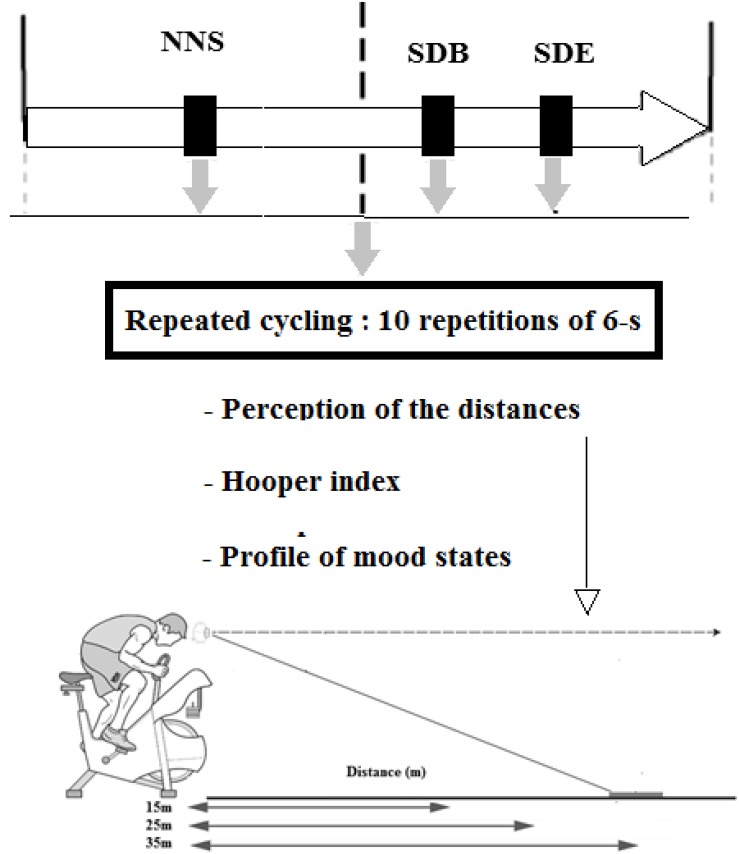
Experimental protocol. NNS, normal sleep night; SDB, sleep deprivation at the beginning of the night; SDE, sleep deprivation at the end of the night.

To examine the effect of PSD and intense effort on distance perception, participants were asked to estimate three egocentric distances, i.e. 15, 25, and 35 m (i.e. only one trial of estimation for each distance), before and after a repeated cycling (RS) exercise and following three conditions of sleep: NNS, PSD at the beginning of the night (SDB), and PSD at the end of the night (SDE) (Figure 1). The three conditions of sleep were performed in a randomized order. The three steps of the experimental protocol were carried out randomly and were separated at least by 1 week. The experiment was performed on vast and unlined ground to avoid perceptual cues and at the same time of the day (i.e. 17h00) to avoid circadian variation of performance (Chtourou and Souissi, 2012). A familiarization session was followed by all participants before the beginning of the test sessions to ensure the synchronization between the physical task (RS) and the cognitive task (distance estimation) when the assistant opened (i.e. immediately at the time when the participant needed to indicate the distance) and closed (i.e. immediately after the indication of the distance by the participant) the curtains, which dissimulates the visual field of the participant. The visual information (i.e. the target is a person) was presented in a soccer field.

### Sleep Analysis

The experimental protocol of sleeping was divided into three different conditions: Condition A: NNS during which participants slept between 22h30 and 06h00, Condition B: SDB during which participants slept from 03h00 to 06h00, and Condition C: SDE during which participants slept from 22h30 to 03h00. Spontaneous body movement was assessed continuously by wrist actigraphy (Actiwatch Sleep & Activity software, version 5.32; Neurotechnology, Cambridge, United Kingdom). Each actigraph contained a piezoelectric transducer sensitive to movements of 1 g acceleration. Actimetric devices were worn on the non-dominant arm from 20h00 of each test session and kept until the end of the experiment. After the end of the experiment, the actigraphs were collected and the data downloaded into a computer via an actigraph interface unit. Data of the actigraphs are presented in Table 1.

**TABLE 1:** Parameters of sleep analysis (mean ± SD, *n* = 10) registered with actimetry during the three sleep conditions [i.e. normal sleep night (NNS), sleep deprivation at the beginning of the night (SDB), and sleep deprivation at the end of the night (SDE)].

Parameters	NNS	SDB	SDE
Start of sleep (h)	23:10 ± 00:32	03:12 ± 00:34	23:15 ± 00:22
End of sleep (h)	07:10 ± 00:42	07:13 ± 00:20	03:13 ± 00:23
Sleep efficiency (%)	90.6 ± 1.4	86.5 ± 1.2*	78.2 ± 1.3*
Sleep latency (min)	41 ± 23	39 ± 20	36 ± 12*
Nocturnal awakening duration (min)	14 ± 2	14 ± 1	14 ± 2

*Significant difference compared to NNS at *p* < 0.05.

#### Egocentric Distance Estimation

Participants were asked to estimate three distances (e.g. 15, 25, and 35 m) before and after RS. In fact, during the protocol, each participant rested a few moments to estimate the distances that separated him from other persons placed at a measured distance. The participant had to tell the distance verbally (i.e. only one attempt) and quickly as soon as he perceived the target person, who would move quickly to another mark for the next estimation. The order of the distances’ presentation (15, 25, and 35 m) was not the same for all participants.

The distances 15, 25, and 35 m were marked by small slates fixed to the ground. The slates were painted in the same color as the ground to make them imperceptible by the participant. Obviously, participants were not informed about the real distances. They were totally focused on the opening of the curtains (i.e. to obscure the vision of the target person for the participant) and the perception of the distance. Participant perception was calibrated before the experiment. Besides, they did not receive feedback that could help them guess the distances. The error of the distance estimation was calculated as: error of the distance (m) = estimated distance (m) − real distance (m).

#### RS Exercise

The repeated cycling exercise was conducted on a friction-loaded cycle ergometer (Monark 894E, Stockholm, Sweden) interfaced with a microcomputer. Prior to each test, participants performed a pretest warm-up consisting of 5 min cycling at 60 W. The RS test consisted of 10 × 6 s maximal cycling against a constant resistance of 50 g kg^–1^ of body mass with a 24 s recovery period after each repetition. Power was calculated for the 10 repetitions as [(rpm × load)/body mass], and the following parameters were then obtained: the peak power (PP), the minimal power (MinP), and the mean power (MP). Moreover, we calculated the total work (the sum of the PP recorded during all repetitions) and the fatigue index: (PP - MinP/MinP) × 100. During exercise, the participants’ visual field was closed by a curtain. Three persons were entrusted to open or close the curtain for 2–3 s.

#### POMS

The mood state of the participants was checked by the POMS questionnaire (McNair et al., 1971) at 3 min after the RS exercise. In fact, participants were asked to describe the intensity of moods they were feeling at that moment (at 06h00 for the SDB, at 03h00 for the SDE, and at 06h00 for the NNS). A list of synonyms was given for each mood state according to McNair et al. (1971). The POMS is a commonly used measure of psychological distress. It distinguishes between seven states of mood: anxiety, depression, anger, confusion, vigor, fatigue, and friendship. The POMS items are rated on a five-point Likert scale ranging from 0 to 4. Responses to each item ranged from 0 to 4, with higher scores indicating negative mood (0 corresponds to “not at all” and 4 corresponds to “extremely”).

#### Hooper Index

According to Hooper et al. (1995), the Hooper Index (HI) is the total of the four subjective ratings, i.e. sleep, stress, fatigue, and muscle soreness [delayed onset muscle soreness (DOMS)], which were collected the day following the game before the recovery training using a psychometric questionnaire (Hooper et al., 1995) to assess the effect of the match on players’ wellness. The questionnaire was composed of four questions relating to perceived quality of sleep during the preceding night, quantity of stress, fatigue, and muscle soreness. Each question was scored separately using subjective rating scales ranging from 1 to 7: from “very very low/good” (i.e. point 1) to “very very high/bad” (i.e. point 7). The HI score is the summation of these four items. The Hooper questionnaire was administered at 2 min after the RS exercise.

### Statistical Analysis

Statistical analyses were done using STATISTICA Software (version 10.1; StatSoft, France). The Shapiro–Wilk *W*-test was used to check normality. A three-way analysis of variance (ANOVA) with repeated measures [3 nights × 2 times distance estimation (pre- and post-effort) × 2 angles] was used to analyze differences between these parameters. A one-way ANOVA with repeated measures (three nights) was used to analyze difference between parameters recorded from the POMS and the Hooper questionnaires and the RS test. If significant main effects were observed, Fisher’s *post hoc* test was performed. Statistical significance was established at *p* < 0.05.

## Results

### Physical Parameters

All measured parameters during RS exercise are presented in Table 2. The results showed a non-significant difference between the three sleeping conditions (i.e. NNS, SDB, and SDE) for PP, MinP, MP, total work, and the fatigue index.

**TABLE 2:** Peak power (PP), minimal power (MinP), mean power (MP), total work, and fatigue index (FI) (mean ± SD, *n* = 10) after repeated cycling during three sleep conditions (i.e. NNS, SDB, and SDE).

Parameters	NNS	SDB	SDE	ANOVA
PP (W kg^–1^)	8.66 ± 0.57	8.36 ± 0.86	8.54 ± 0.84	*F*(2,18) = 0.6; *p* = 0.559
MinP (W kg^–1^)	6.29 ± 0.90	6.60 ± 0.48	6.39 ± 0.81	*F*(2,18) = 0.87; *p* = 0.437
MP (W kg^–1^)	7.16 ± 0.63	7.35 ± 0.59	7.31 ± 0.68	*F*(2,18) = 0.81; *p* = 0.46
Total work (W kg^–1^)	71.60 ± 6.35	73.53 ± 5.87	73.12 ± 6.77	*F*(2,18) = 0.81; *p* = 0.46
FI (%)	40.79 ± 25.98	26.94 ± 12.08	34.91 ± 16.86	*F*(2,18) = 1.55; *p* = 0.239

### HI

The data showed that sleep, fatigue, stress, DOMS and HI were significantly lower after NNS compared to SDB and SDE (*p* < 0.05; Table 3).

**TABLE 3:** Sleep, fatigue, stress, and delayed onset muscle soreness (DOMS) (mean ± SD, *n* = 10) after three sleep conditions (i.e. NNS, SDB, and SDE).

Parameters	NNS	SDB	SDE	ANOVA
Sleep (AU)	3.0 ± 1.2	5.3 ± 1.2***	5.2 ± 1.2***	*F*(2,18) = 18.04; *p* < 0.001
Fatigue (AU)	2.4 ± 0.8	4.3 ± 1.3***	3.9 ± 1.4**	*F*(2,18) = 9.37; *p* = 0.002
Stress (AU)	2.3 ± 0.7	4.4 ± 1.2***	3.6 ± 1.5*	*F*(2,18) = 7.92; *p* = 0.003
DOMS (AU)	1.9 ± 0.7	4.1 ± 1.2***	3.5 ± 1.6**	*F*(2,18) = 11.19; *p* < 0.001

*^∗^, ^∗∗^, and ^∗∗∗^ denote significant difference from NNS at *p* < 0.05, *p* < 0.01, and *p* < 0.001, respectively. AU, arbitrary unit.*

### POMS

Table 4 displays the score of POMS registered after NNS, SDB, and SDE. A significant difference of the POMS scores between NNS and both SDB (*p* < 0.01) and SDE (*p* < 0.05) was demonstrated. However, no significant difference of the POMS score was observed between SDB and SDE.

**TABLE 4:** Profile of mood states (POMS) questionnaire after the sleep conditions (i.e. NNS, SDB, and SDE).

Parameters	NNS	SDB	SDE	ANOVA
Anxiety (AU)	4.5 ± 3.7	8 ± 4.7	6.7 ± 5.3	*F*(2,18) = 3; *p* = 0.075
Anger (AU)	4.6 ± 5.3	9.2 ± 8.1	7.1 ± 8.9	*F*(2,18) = 2.52; *p* = 0.108
Confusion (AU)	4.6 ± 3.8	8.6 ± 5.1*	6.7 ± 4.5	*F*(2,18) = 4.08; *p* = 0.035
Depression (AU)	2.9 ± 4	7.2 ± 7.4	5.3 ± 7.6	*F*(2,18) = 2.41; *p* = 0.118
Fatigue (AU)	4.3 ± 4.1	5.9 ± 4.6	5.3 ± 4.6	*F*(2,18) = 0.72; *p* = 0.502
Vigor (AU)	13.7 ± 7.1	5.4 ± 4.2**	5.4 ± 4.2**	*F*(2,18) = 8.14; *p* = 0.003
Interpersonal relationships (AU)	14.1 ± 7.9	7.2 ± 3.7**	7.2 ± 3.7**	*F*(2,18) = 7.3; *p* = 0.005
POMS (AU)	7.2 ± 16.1	33.5 ± 26.2**	25.7 ± 29.2*	*F*(2,18) = 6.25; *p* = 0.009

* and ** denote significant difference from NNS at *p* < 0.05, *p* < 0.01, and *p* < 0.001.

### Effect of RS Exercise on Distance Perception After Sleep Deprivation

#### 35 m Distance

The statistical analysis showed significant main effects for sleep (*F* = 6.63, *p* < 0.01, $\eta_{\text{p}}^{2}$ = 0.42), effort (*F* = 10.25, *p* < 0.05, $\eta_{\text{p}}^{2}$ = 0.53), and angle of view (*F* = 36.54, *p* < 0.001, $\eta_{\text{p}}^{2}$ = 0.80). However, no significant interaction was observed. The *post hoc* test showed that the estimation of the distance was better after NNS compared to SDB and SDE for the front and the two side angles (*p* < 0.05) (Table 5). The estimation of the distance was also better after RS in comparison with before RS for the side angle during NNS and SDE (*p* < 0.05). For the angle of view, the estimation of the distance was better for the front compared to the side angle both before and after the RS for the two PSD conditions (*p* < 0.05). However, after NNS, the estimation of the distance was better for the two side angles compared to the front after RS (*p* < 0.05). Likewise, the estimation of the distance was better after NNS compared to SDB and SDE for the front and the two side angles (*p* < 0.05).

**TABLE 5:** The error estimations of 35 m according to the real distance (mean ± SD, *n* = 10) after NNS, SDB, and SDE before and after exercise.

	Before exercise			After exercise		
	Side	Face	Mean	Side	Face	Mean
NNS	−2.5 ± 5.7	2.5 ± 8.2^#^	0 ± 6.6	−0.2 ± 7.1^[*dollar*]^	4 ± 5.7^#^	1.9 ± 6.1
SDB	−8.5 ± 6.1*	−5.1 ± 7.1*^#^	−6.8 ± 6.5	−7.3 ± 7*	−4.3 ± 8*^#^	−5.8 ± 7.3
SDE	−8.1 ± 63*	−3.5 ± 5.6*^#^	−5.8 ± 5.9	−5.5 ± 6.5*^[*dollar*]^	−3.1 ± 5.3*^#^	−4.3 ± 5.5

*, significantly different from NNS at *p* < 0.05; $, significantly different from before effort at *p* < 0.05; and #, significantly different from side at *p* < 0.05.

#### 25 m Distance

The analysis showed significant main effects for effort (*F* = 27.96, *p* < 0.001, $\eta_{\text{p}}^{2}$ = 0.61) and angle of view (*F* = 14.55, *p* < 0.01, $\eta_{\text{p}}^{2}$ = 0.61). However, the analysis showed no significant main effects for sleep (*F* = 1.88, *p* > 0.05, $\eta_{\text{p}}^{2}$ = 0.17). Likewise, no significant interaction was observed. As regards the effect of sleep deprivation, the *post hoc* test showed that the distance estimation was better after NNS in comparison to SDB and SED for the front angle (*p* < 0.05) (Table 6). Concerning the effect of effort, the distance estimation was better after compared to before RS for the front angle after NNS (*p* < 0.05). For the angle of view, the estimation of the distance was better from the front compared to the two side angles after RS after NNS (*p* < 0.05).

**TABLE 6:** The error estimations of 25 m (mean ± SD, *n* = 10) according to the real distance after NNS, SDB, and SDE before and after exercise.

	Before exercise			After exercise		
	Side	Face	Mean	Side	Face	Mean
NNS	−3.6 ± 4.1	−2.5 ± 4.9	−3 ± 3.3	−2.9 ± 4.2	1.2 ± 7.5^$#^	−0.8 ± 5.5
SDB	−6.2 ± 4.4	−4.2 ± 5.6	−5.2 ± 4.8	−5.1 ± 5.6	−5.5 ± 5.1*	−5.3 ± 5.2
SDE	−6.3 ± 4.2	−4.1 ± 5.2	−5.2 ± 4.3	−3.5 ± 4.7	−2.2 ± 4.6*	−2.9 ± 4.5

*, significantly different from NNS at *p* < 0.05; $, significantly different from before effort at *p* < 0.05; and #, significantly different from side at *p* < 0.05.

#### 15 m Distance

The analysis showed no significant main effects for sleep (*F* = 2.8, *p* > 0.05, $\eta_{\text{p}}^{2}$ = 0.23), effort (*F* = 0.02, *p* > 0.05, $\eta_{\text{p}}^{2}$ = 0.002), and angle of view (*F* = 0.003, *p* > 0.05, $\eta_{\text{p}}^{2}$ = 0.003). Likewise, no significant interaction was observed. Concerning the effect of sleep deprivation, the statistical analysis showed that the estimation of the distance was better after NNS in comparison with SDE for the two side angles after RS (*p* > 0.05) (Table 7). The results showed, also, that the distance estimation was better after NNS than SDB and SDE for the front and both side angles (*p* < 0.05) after RS.

**TABLE 7:** The error estimations of 15 m (mean ± SD, *n* = 10) according to the real distance after NNS, SDB, and SDE before and after exercise.

	Before exercise			After exercise		
	Side	Face	Mean	Side	Face	Mean
NNS	−1.1 ± 3.6	−1.1 ± 6.3	−1.1 ± 4.8	−1 ± 4.6	−0.4 ± 4.3	−0.7 ± 3.9
SDB	−2.8 ± 4.4	−3.2 ± 3.8	−3 ± 3.8	−3.5 ± 4.1*	−3.6 ± 4.3*	−3.5 ± 4
SDE	−4.1 ± 1.7*	−3.3 ± 2.9	−3.7 ± 2.2	−3.5 ± 2.1*	−4.1 ± 1.7*	−3.8 ± 1.7

*, significantly different from NNS at *p* < 0.05.

## Discussion

The purpose of the present study was to explore the effect of PSD on egocentric distance estimation before and after an exhaustive exercise in soccer players. We demonstrated that distance estimation was affected by PSD following an intense effort. In fact, all distances (i.e. 15, 25, and 35 m) were underestimated after SDB and SDE at rest and after RS. Moreover, the results showed that the estimation of the distance was more affected after SDB than after SDE.

The results of the present study showed no effect of SDB on the RS, which corroborates Mougin et al. (1996), who reported the absence of any SDB effect on short-term maximal performance. Also, the result showed no significant difference between NNS and SDE on RS performances. These results support those of Taheri and Arabameri (2012), who observed no significant alterations in anaerobic performance resulting from one night of sleep loss. Nevertheless, others studies showed that anaerobic performance decreased at the end of the day after SDE (Abedelmalek et al., 2013a, b; HajSalem et al., 2013).

It is sure that sleep deprivation influences visual perception (Kong et al., 2011). Indeed, a residual visual processing capacity can be exhausted by an increasing perceptual load (Kong et al., 2011). The magnitude of the repetition suppression to unattended scenes may be used to track the visual processing capacity (Kong et al., 2011).

Otherwise, the negative effects on perception could be due to sleep deprivation and other sleep disturbances induced during the experiments, such as the social jet lag that occurred due to the change in the sleep and wake times. Social jet lag refers to a change in sleeping hours, for example, from workdays to non-working days (Wittmann et al., 2006; Simmons et al., 2015; Malhotra, 2017), and has been reported to affect postural control (Umemura et al., 2018). Also, jet lag is the term applied to a group of symptoms that exist transiently after undergoing a rapid time-zone transition, and it has been reported that this situation negatively affects physical and cognitive performance (Davenne, 2009; Edwards and Waterhouse, 2009).

The difference of the duration of sleep deprivation between experiments and the difference between the standardized physiological tests used can explain the discrepancies between results. At rest and following SDB and SDE, the results showed an underestimation of all distances. In fact, the participants compressed the real distances even at rest or after RS, which could be explained by the impairment of visual perception (Thomas et al., 2000) and an unreached activation level of the central nervous system, which didn’t allow participants to efficiently estimate distances and then reach precise and pertinent cognitive performances (Arcelin et al., 1998; Jarraya et al., 2013). Thus, in this lexicon, it is noticeable that this phenomenon had to affect the capability of the participant to perceive targets (Kong et al., 2011).

The underestimation found could also be due to the deterioration of mood state and psychometric and cognitive performances (Pilcher and Huffcutt, 1996). In this context, the alteration of neuropsychological functions such as verbal immediate memory, vigilance (Fulda and Schulz, 2001), attention, and reaction time (Jarraya et al., 2014) could also explain this underestimation. Consistently with previous studies (Davenne, 2009; Goel et al., 2013), the results showed a decline of all mood variables especially after SDB compared to SDE. An increased level of anxiety, anger, confusion, stress, and fatigue after sleep loss has been previously reported (Blumert et al., 2007). A previous study reported that these parameters induced peripheral and central fatigue, which could induce a deterioration of perceived distances and, thus, a decline of cognitive performance (Davenne, 2009). Accordingly, Goel et al. (2013) have previously demonstrated that sleep deprivation impairs visual short-term memory and limits its capacity. Likewise, Chee et al. (2006) showed that involved parametrically manipulated perceptual or memory load in two visual tasks after sleep deprivation decreases behavioral performance and reduces parietal and extra-striatal activation.

In the present study, the underestimation was significantly higher when the distance was larger (35 vs. 25 and 15 m). Thus, the higher the perceived distance is, the bigger the error of the estimation will be, and accordingly, the underestimation will be greater (Gilinsky, 1951). It is commonly assumed that perceptual space is compressed, leading to a large distance underestimation (Hecht et al., 1999). In addition, the notion of sagittal compression in action space (between 2 and 30 m) is consistent with findings that slope, or geographical slant, is overestimated at distances up to 30 m (Proffitt et al., 1995). On the other hand, the distance estimation is more accurate within personal space (a distance <2 m), where convergence and retinal disparity are informative. Moreover, the aerial perspective also suggests that the atmosphere reduces the visibility of faraway objects, which points out that systematic errors in distance occur in distance judgments (Roscoe, 1984).

Rather than optical units, distance can be coded in effort units, which means that perceiving egocentric distance is not only a function of the optical variables but also a function of people’s physiological potential to act (Witt et al., 2004). Thus, the further the distance is, the more effort to act it requires, and the more likely it is to be misestimated.

The data of the present study revealed that egocentric distance estimation was affected by SDB more than SDE. Jarraya et al. (2014) concluded that SDB had a greater effect on cognitive performances than SDE. This finding could be attributable to the absence of the third and fourth periods of sleep, commonly called deep sleep (Davenne, 2009), which is essential for human physical and mental health as it consolidates memory (Peigneux et al., 2001; Rauchs et al., 2005) and enhances physiological processes such as luteinizing hormone secretion, especially during late childhood (Shaw et al., 2015).

The population profile could explain in part why the participants had a tendency to highly underestimate distances after SDB than SDE.

According to Horne Ostberg’ questionnaire (1976), participants were classified as morningness (i.e. early lark). The findings of the present study corroborate those of Mongrain et al. (2008), who showed that the differences in neurobehavioral responses after the fragmentation of sleep can be explained by the participant chronotype. Using four different measuring tests (VAS: visual analog scale, PVT: psychomotor vigilance task, EEG: awakening recorder, and MSLT: sleep latency test), Mongrain et al. (2008) observed higher levels of subjective alertness among evening-type and even morning-type participants during the day following the recovery night compared to NNS. The results of the VAS and the electroencephalography (EEG) revealed better recovery after fragmentation and recovery topics for the evening types as opposed to morning types, while PVT and MSLT showed an opposite result, with better recovery for morning-type participants. However, morning types recover their vigilance levels more quickly than evening types. Based on this latter experiment, the present study concluded that the difference of neurobehavioral responses after fragmentation and recovery sleep is attributable to the participant chronotype. In addition to the morning/evening types and their relation to sleep deprivation deficit, actigraphy parameters were measured in order to track the variance of sleep (Forner-Cordero et al., 2018). The results have reported a decrease in sleep efficiency and sleep latency parameters during SDB and SDE compared to NNS.

The significant underestimation of all distances (i.e. 15, 2, and 35 m) after RS following SDB and SDE compared to NNS could be associated to the activation level of the nervous system (Horgervost et al., 1996). Likewise, the results of the present study showed that the estimation of the distance was better during NNS compared to SDB and SDE for the front and the two side angles. It is certain that a lack of sleep would compound the disruption of vision, which is observed by a negative effect on the estimated distance from different angles (0° vs. 45°), more specifically from the side view angle, which is further amplified with exercise. Thus, the observed results could be explained by the combined effect of sleep deprivation, the angle of view, and effort. Moreover, the change in the perception of the distance could be due to modifications of the plasma level of the hormones involved in stress and waking, e.g. catecholamines, dopamine, and cortisol, that stimulate alertness and vigilance during exercise (Horgervost et al., 1996). Nevertheless, the lack of biochemical measurement was one of the limitations of the current study, and further studies including measurements of catecholamines (i.e. adrenaline and noradrenalin) are needed to better clarify this point.

Further research is required to confirm what dimensions of distance estimation for different angles of vision are affected by sleep loss, especially with a focus on RS exercise. Also, the extrapolation of assumptions regarding sleep and estimation of distance in different angles of vision is yet to be understood. From a scientific perspective, it is pertinent that some factors should be considered in future studies when defining the effect of sleep on distance estimation in different angles of vision, especially during effort, including more analyses such as sleep patterns and the physiology of the ocular motor system.

## Conclusion

In conclusion, SDB and SDE affect egocentric distance estimation either at rest or after exercise. Likewise, error estimation was relatively higher after SDB than SDE either before or after effort in soccer players.

### Practical Recommendations

• Athletes must take into consideration the visual angle as a determinant of the estimation of the distance, especially after sleep loss.
• If problems of sleep loss persist, these should be dealt with using adequate strategies.

## Data Availability Statement

The datasets generated for this study are available on request to the corresponding author.

## Ethics Statement

The studies involving human participants were reviewed and approved by the Ethics Committee of the University of Sfax, Tunisia. The patients/participants provided their written informed consent to participate in this study.

## Author Contributions

HB, MJ, HC, and WM conceived the study. HB, MJ, HC, WM, PN, TR, and BK designed the study, revised the manuscript, and approved the final version of the manuscript. HB collected the data, analyzed and interpreted the data, and drafted the manuscript.

## Conflict of Interest

The authors declare that the research was conducted in the absence of any commercial or financial relationships that could be construed as a potential conflict of interest.
